# A multiscale active structural model of the arterial wall accounting for smooth muscle dynamics

**DOI:** 10.1098/rsif.2017.0732

**Published:** 2018-02-07

**Authors:** Alberto Coccarelli, David Hughes Edwards, Ankush Aggarwal, Perumal Nithiarasu, Dimitris Parthimos

**Affiliations:** 1 Division of Cancer and Genetics, School of Medicine, Cardiff University, Cardiff, UK; 2 Institute of Life Sciences, Medical School, Swansea University, Swansea, UK; 3 Zienkiewicz Centre for Computational Engineering, College of Engineering, Swansea University, Swansea, UK

**Keywords:** media layer, Ca^2+^ dynamics, smooth muscle, multiscale modelling

## Abstract

Arterial wall dynamics arise from the synergy of passive mechano-elastic properties of the vascular tissue and the active contractile behaviour of smooth muscle cells (SMCs) that form the media layer of vessels. We have developed a computational framework that incorporates both these components to account for vascular responses to mechanical and pharmacological stimuli. To validate the proposed framework and demonstrate its potential for testing hypotheses on the pathogenesis of vascular disease, we have employed a number of pharmacological probes that modulate the arterial wall contractile machinery by selectively inhibiting a range of intracellular signalling pathways. Experimental probes used on ring segments from the rabbit central ear artery are: phenylephrine, a selective *α*1-adrenergic receptor agonist that induces vasoconstriction; cyclopiazonic acid (CPA), a specific inhibitor of sarcoplasmic/endoplasmic reticulum Ca^2+^-ATPase; and ryanodine, a diterpenoid that modulates Ca^2+^ release from the sarcoplasmic reticulum. These interventions were able to delineate the role of membrane versus intracellular signalling, previously identified as main factors in smooth muscle contraction and the generation of vessel tone. Each SMC was modelled by a system of nonlinear differential equations that account for intracellular ionic signalling, and in particular Ca^2+^ dynamics. Cytosolic Ca^2+^ concentrations formed the catalytic input to a cross-bridge kinetics model. Contractile output from these cellular components forms the input to the finite-element model of the arterial rings under isometric conditions that reproduces the experimental conditions. The model does not account for the role of the endothelium, as the nitric oxide production was suppressed by the action of L-NAME, and also due to the absence of shear stress on the arterial ring, as the experimental set-up did not involve flow. Simulations generated by the integrated model closely matched experimental observations qualitatively, as well as quantitatively within a range of physiological parametric values. The model also illustrated how increased intercellular coupling led to smooth muscle coordination and the genesis of vascular tone.

## 1. Introduction

The arterial wall can be viewed as a control system able to regulate blood flow in order to satisfy local tissue oxygenation and nutrition requirements. This function is performed, in part, by spontaneous fluctuations in vascular tone and diameter, known as vasomotion, and is facilitated by the contractile apparatus located within the smooth muscle layer of the arterial wall [1]. Arteries form an anisotropic structure composed of three primary layers that perform distinct functions. The outermost layer, adventitia, is made of a tissue with fibre dispersion which confines the inner arterial structures. Active vascular contractility is governed by the smooth muscle cells (SMCs) located in the second layer, called media. The endothelium, a cellular mono-layer lining the inner surface of the blood vessels, operates as an active interface between the blood flow and the medial layer. Between these structures lie layers of artery-specific connective tissue called elastic laminae [2], which largely convey the mechano-elastic properties of the vascular wall. The active contractile machinery of SMCs, driven by the phosphorylation of the actin–myosin motors, is catalysed by intracellular Ca^2+^. Under physiological conditions the intracellular Ca^2+^ concentration exhibits modest variations, however, when operative conditions deviate far from normal, such as in the case of injury or under pharmacological interventions, significant fluctuations in intracellular Ca^2+^ concentrations may occur that are reflected in the pronounced dynamical variation in vascular tone [3–5]. The complex structure and function of the arterial wall, even in the absence of blood flow, suggest that a mathematical/computational multi-physics approach is necessary in order to elucidate the dynamical intricacy of the underlying biological system and to address questions that so far evade experimental investigations. To address this problem, a considerable number of multiscale/multi-component models for the arterial wall have been proposed in recent years [6–9]. The development of models accounting for the elastic behaviour of the vascular wall has been based on extensive experimentation on the mechanical properties of vascular tissue under a variety of stress–strain conditions [10–14]. Based on these findings, several methodologies have been proposed in the last decade for simulating the smooth muscle contractility [15–20]. In spite of considerable advances, the active component of vascular contractility, centred on the cellular Ca^2+^ dynamics of the smooth muscle has not been incorporated in a systematic way. This is particularly important as, according to classification by Fischer [21], the smooth muscle responsible for arterial vasomotion can be considered of the ‘fast type’ from a mechano-elastic point of view, and is therefore markedly sensitive to the cellular wall dynamics. Detailed modelling of vasomotion as an expression of multi-channel ionic signalling that regulates arterial smooth muscle Ca^2+^ dynamics, has been proposed in [22,23]. This work was extensively validated against a broad range of pharmacological interventions that specifically inhibit individual transport mechanisms. Extended cellular arrays of coupled SMCs were subsequently used to study the emergence of large-scale synchronization [24]. In this work, we have employed a hybrid model, based on [22,23], to integrate the active contractile behaviour of the media smooth muscle layer with the structural response of the arterial wall. The computational model developed incorporates two distinct scales: the cellular, where cytosolic Ca^2+^ catalyses cross-bridge (CB) kinetics, and the continuous where the contractile units (CUs), and therefore the arterial tissue, exhibit deformation and stress. Evaluation of the CB kinetics at cellular level relies on a modified version of the Hai & Murphy model [25,26]. The cellular network finite-element design follows the anatomical morphology of the tissue considered. For the mechano-elastic characterization of the arterial wall, we follow the work of [17,19,20]. From the structural point of view, the tissue is assumed to be a fibre-reinforced hyper-elastic material [11,27] and incompressibility is enforced by means of a standard penalty method. Simulation results were validated against experimental recordings of vascular tone obtained from rabbit ear arteries under isometric conditions. In the absence of fluid flow, the relationship between stress and deformation of the vascular structure becomes the only stress/deformation-generator mechanism. In addition, we were able to minimize the influence of the endothelium on the smooth muscle contractile apparatus by inhibiting production of nitric oxide (NO), the dominant endothelial control mechanism in the central rabbit ear artery [28]. The performance of the modelling framework has been tested against a number of cellular Ca^2+^ dynamics scenarios, induced by drug interventions able to specifically modulate the SMCs contractile machinery.

## 2. Modelling methodology

**Figure 1.**
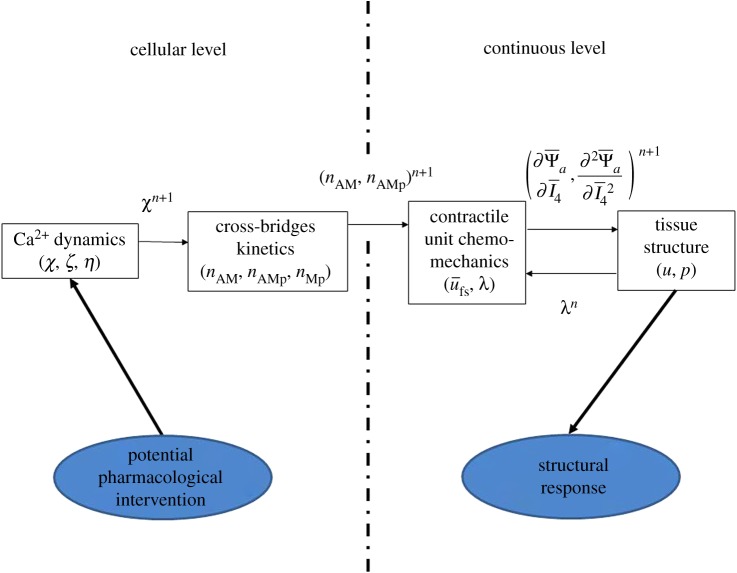
Levels and subsystems of the media multiscale model. Thin arrows indicate quantities transmitted between the subsystems. Variables in the boxes are internal variables of the subsystems. Thick arrows represent the input and output of the model. (Online version in colour.)

The multiscale modelling methodology that accounts for the contractile dynamics of the arterial wall is presented in figure 1, where the flow of information is highlighted. The model developed is based on the coupling of the smooth-muscle contractile apparatus with the mechano-elastic properties of the arterial wall. We outline here the mathematical formulation of the model components and their interface as a requirement for emergent coupled behaviour. At the cellular level, Ca^2+^ dynamics is described by a system of three coupled nonlinear ordinary differential equations which express cytosolic Ca^2+^ concentration (*χ*), Ca^2+^ concentration within the sarcoplasmic reticulum (SR) (*ζ*) and the membrane potential (*η*). Physiological regulation of these variables is due to the coordinated effect of multiple ionic currents that have been incorporated in the formulation of the model and are described in detail in the following section. Cytosolic Ca^2+^ is the primary catalyst for the phosphorylation of CB formation that occurs within each SMC, and which facilitates the contractile response of the cellular cytoskeleton. At the level of the CU, four distinct states can represent the CB kinetics: dephosphorylated myosin (*n*_M_), phosphorylated myosin (*n*_Mp_), attached actin–myosin filaments (*n*_AM_) and attached phosphorylated actin–myosin filaments (*n*_AMp_). The model assumes a spatial uniformity of CB within the cytosol. The state describing the fractions of attached actin–myosin filaments (represented as *n*_AM_+*n*_AMp_), along with variable *λ* that describes the physical deformation of the contractile elements (the stretch ratio in the tissue structure model), serve as inputs to the CU model, where the active free energy function is computed. The calculations performed in the CU model depend on the internal variable 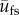, which represents the current relative sliding between the filaments. The derivatives of the active free energy function with respect to the fourth invariant (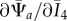, 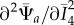), calculated at the CU subsystem, are then passed directly to the tissue structural mechanics model (with displacement *u* and pressure *p* as variables), where the total (passive and active) tissue stress and deformation is evaluated. This accounts for the arterial wall structural response. Several stages of this physiological process may be probed through targeted pharmacological interventions (e.g. specific ion channel blockers) to reproduce and investigate the origin of vascular disease states. All the model parameters are listed in tables 1, 2 and 4.

**Table 1.** Table of parameters: Ca^2+^ dynamics and cross-bridges (CB) kinetics. Parametric values associated with Ca^2+^ dynamics are taken from Boileau *et al*. [29], while parameters for CB kinetics are from Parthimos *et al*. [22].

parameter	description	value
Ca^2+^ dynamics		
*Φ*_*A*_	Ca^2+^ influx via NSCC	0.6 (μM s^−1^)
*L*_SR_	SR leak rate constant	0.025 (s^−1^)
*γ*	scaling factor	1.0 (V μM^−1^)
*A*_S_	SOCC parameter	0.0 (s^−1^)
*ζ*_S_	SOCC parameter	4.0 (μM )
*E*_Ca_	VOCC influx cell conductance	12.0 (μM (V s)^−1^)
*z*_Ca1_	VOCC influx reversal potential	0.13 (V)
*z*_Ca2_	VOCC influx half-point of activation sigmoid	−0.024 (V)
*R*_Ca_	VOCC influx max. slope of activation sigmoid	0.0085 (V)
*E*_NCX_	NCX cell conductance	43.8 (μM (V s)^−1^)
*z*_NCX_	NCX reversal potential	−0.04 (V)
*x*_NCX_	NCX half-point of Ca^2+^activation	0.5 (μM)
*B*_SR_	SR uptake rate	400.0 (μM s^−1^)
*x*_SR_	SR uptake half-point of ATPase activation sigmoid	4.4 (μM)
*n*_SR_	SR uptake Hill coefficient	2 (−)
*C*_Ry_	RyR CICR rate	1250.0 (μM s^−1^)
*y*_Ry_	RyR CICR half-point of Ca^2+^ efflux sigmoid	8.9 (μM)
*x*_Ry_	RyR CICR half-point of CICR activation sigmoid	0.9 (μM)
*m*_Ry_	RyR CICR Hill coefficient	2 (−)
*p*_Ry_	RyR CICR Hill coefficient	4 (−)
*D*_EX_	Ca^2+^ extrusion by ATPase pump rate	6.25 (μM s^− 1^)
*z*_Ex_	Ca^2+^ extrusion by ATPase pump constant	−0.1 (V)
*R*_Ex_	Ca^2+^ extrusion by ATPase pump constant	0.25 (V)
*k*_Ex_	Ca^2+^ extrusion by ATPase pump constant	2
*E*_Cl_	Cl^−^ channels cell conductance	65.0 (μM (V s)^−1^)
*z*_Cl_	Cl^−^ channels reverse potential	−0.025 (V)
*x*_Cl_	Cl^−^ channels Ca^2+^ sensitivity	0.0 (μM)
*E*_K_	K^+^ efflux cell conductance	43.0 (μM s^−1^)
*z*_K_	K^+^ efflux reverse potential	−0.095 (V)
*z*_Ca3_	K^+^ efflux half-point of activation sigmoid	−0.027 (V)
*R*_K_	K^+^ efflux max. slope of K_Ca_ activation sigmoid	0.012 (V)
*β*_K_	K^+^ efflux Ca^2+^ sensitivity of K_Ca_ channel activation sigmoid	0.0 (μM)
*α*_C_	cellular Ca^2+^ diffusivity	1.0 (s^−1^)
*α*_V_	cellular voltage diffusivity	1.0 (s^−1^)
CB kinetics		
*τ*_0_	kinetic model fitting parameter	1.7 (s^−1^)
*τ*_2_	kinetic rate	0.5 (s^−1^)
*τ*_3_	kinetic rate	0.4 (s^−1^)
*τ*_4_	kinetic rate	0.1 (s^−1^)
*τ*_5_	kinetic rate	1.0 (s^−1^)
*χ*_0_	kinetic saturation constant	0.6 (μM)
*θ*	enhancement kinetics coefficient	1.0 (−)

**Table 2.** Table of parameters: contractile units mechanics and tissue structure. All parametric values reported in the table are from Murtada & Holzapfel [20].

parameter	description	value
CU mechanics		
*α*_*a*_	material parameter	26.68 (kPa)
*β*_*a*_	material parameter	0.00833 (s^−1^)
*κ*_AMp_	parameter related to the force of a power-stroke of a single CB	203.71 (kPa)
*κ*_AM_	parameter related to the force-bearing capacity of a dephosphorylated CB during muscle extension	61.14 (kPa)
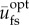	material parameter	0.48 (−)
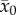	material parameter	0.4255 (−)
tissue structure		
*κ*	bulk modulus	4.0 (kPa)
*μ*_a_	active shear modulus	5301.0 (kPa)
*μ*_p_	passive shear modulus	0.84 (kPa)
*c*_p1_	material parameters	3.15 (kPa)
*c*_p2_	material parameter	0.035 (−)

### 2.1. Cellular level

#### 2.1.1. Cytosolic Ca^2+^ homeostasis

**Figure 2.**
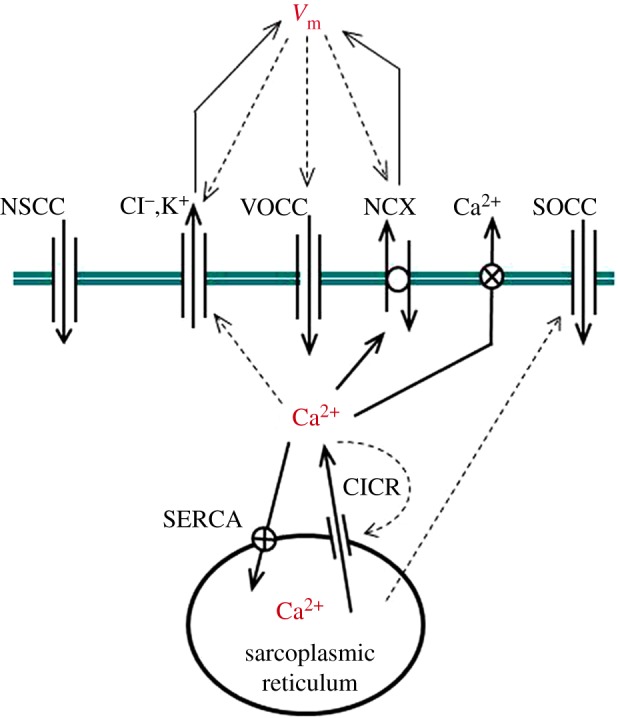
Cellular Ca^2+^ model illustrating the membrane and intracellular components. (Online version in colour.)

The fundamental processes involved in the regulation of cytosolic Ca^2+^ concentrations are outlined in figure 2. All the ionic pathway contributions depicted have been incorporated in a model that combines the approaches previously proposed in [22,23]. In particular, we employed a system of three ordinary differential equations, as in [23], but maintained the predominance of ryanodine-sensitive over InsP3-sensitive intracellular stores, as appropriate for the rabbit ear arteries used in the experimental study [22]. Moreover, the model constructed maintains the well-established view that smooth muscle Ca^2+^ dynamics are due to the interplay of a membrane and an intracellular oscillator [30]. The mechanism underlying the intracellular Ca^2+^ oscillator is Ca^2+^ induced-Ca^2+^ release (CICR) via the ryanodine receptor. Once released into the cytosol, Ca^2+^ is re-sequestered by the stores through the sarcoplasmic/endoplasmic reticulum ATPase (SERCA), thus completing the cycle. By contrast, the membrane oscillator is centred on cellular polarization and voltage-operated Ca^2+^ channels (VOCCs). Membrane potential is mainly influenced by the balance between K^+^ and Cl^−^ gradients, and by Na^+^-Ca^2+^ exchange (NCX), depending on whether the exchanger operates in forward or reverse mode. The current model also includes the store operated Ca^2+^ channel (SOCC), which links directly store content with cytosolic Ca^2+^ influx [31]. This addition allows for testing theoretically the role of this channel in specific pharmacological intervention scenarios. An explicit delineation of the modelling components is presented below.

##### 2.1.1.1. Ionic channels

The extracellular influx is the sum of the concentration currents *Φ*_*A*_, *Φ*_S_, *Φ*_V_ and *Φ*_N_, which represent the Ca^2+^ permeable non-selective cation channels (NSCC), SOCC, voltage-operated cation channels (VOCC) and reverse mode Na^+^-Ca^2+^ exchange (NCX), respectively. In this study, the first current is assumed to be constant, while the others depends on *χ* and *η* via the following equations:
2.1
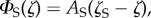

2.2
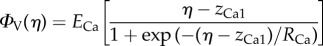

2.3
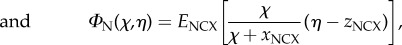
where *A*_S_, *ζ*_S_, *E*_Ca_, *z*_Ca1_, *R*_Ca_, *E*_NCX_, *x*_NCX_ and *z*_NCX_ are cellular model parameters. The SR acts as an inner store, uptaking cytosolic Ca^2+^ by means of the SERCA pump (*Φ*_B_(*χ*)) and releasing it into the cytosol through ryanodine-sensitive SR-Ca^2+^ release channel (*Φ*_C_(*χ*, *ζ*)). Store leakage (*Φ*_L_(*χ*, *ζ*)) is also accounted for. These fluxes are described through the following expressions:
2.4
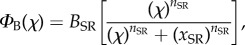

2.5
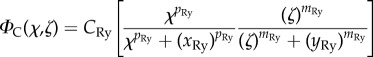

2.6
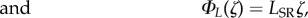
where *B*_SR_, *n*_SR_, *x*_SR_, *C*_Ry_, *p*_Ry_, *m*_Ry_, *x*_Ry_, *y*_Ry_ and *L*_SR_ are cellular model constants. The Ca^2+^ extrusion from the cytosol by ATPase pump (*Φ*_D_(*χ*, *η*)) is modelled as follows:
2.7
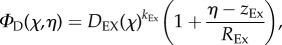
where *D*_EX_, *k*_EX_, *z*_EX_ and *R*_EX_ are cellular model parameters. The chloride (*Φ*_Cl_(*χ*, *η*)) and potassium (*Φ*_K_(*χ*, *η*)) ion membrane fluxes are modelled as follows:
2.8
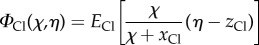
and
2.9
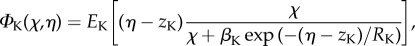
where *E*_Cl_, *x*_Cl_, *z*_Cl_, *E*_K_, *z*_K_, *β*_K_ and *R*_K_ are cellular model constants.

##### 2.1.1.2. Intercellular communication

In a cellular cluster, each cell is able to communicate with its neighbours by exchanging Ca^2+^ ion (*J*_Ca_) and voltage (*J*_V_) currents. Assuming that the cellular size (ratio between the volume and surface) is uniform along the grid, we can define the net fluxes 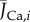 and 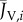 exchanged by the *i*th cell as follows:
2.10
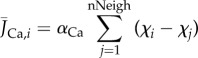
and
2.11
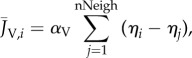
where nNeigh is the number of adjacent elements, and 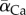 and 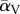 are the intercellular diffusion coefficients.

##### 2.1.1.3. Global cellular balance

The system variables *χ*, *ζ* and *η* evolve in time according to the following system of nonlinear ordinary differential equations:
2.12
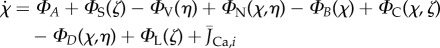

2.13
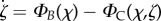

2.14
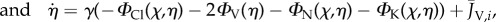
where *γ* is a scaling factor relating the net movement of ion fluxes to the membrane potential.

#### 2.1.2. Cross-bridges kinetics

**Figure 3.**
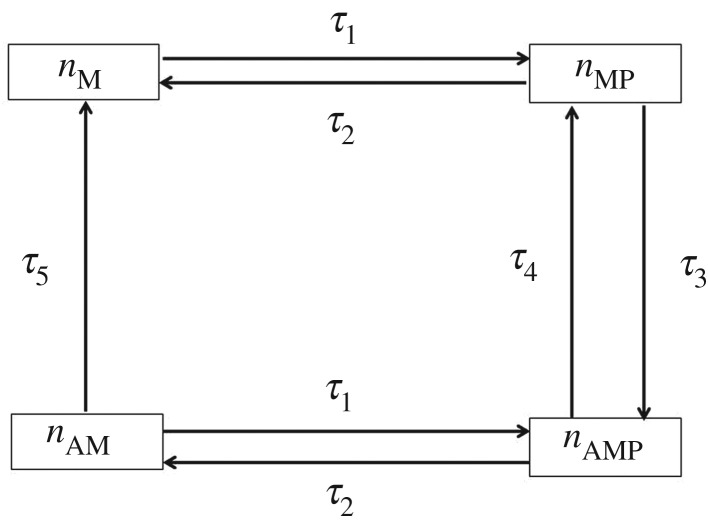
Four states of the actin–myosin cross-bridges kinetics. Each arrow represents a transition between two states and is associated with a specific kinetic rate. The arrows indicate the direction of the transitions.

The kinetics is described through four different states representing the fraction of CB that are: (i) attached and dephosphorylated (*n*_AM_), (ii) attached and phosphorylated (*n*_AMp_), (iii) detached and dephosphorylated (*n*_*A*_) and (iv) detached and phosphorylated (*n*_Ap_). The temporal evolution of the four-state kinetics shown in figure 3 is described through the following system of ordinary differential equations:
2.15
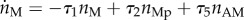

2.16
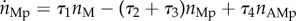

2.17
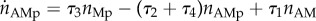

2.18
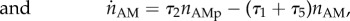
where *τ*_1_, *τ*_2_, *τ*_3_, *τ*_4_ and *τ*_5_ are the kinetic rates. Among these, *τ*_1_, depends on the intracellular Ca^2+^ concentration (*χ*) via [22]:
2.19
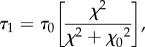
where *τ*_0_ and *χ*_0_ are the material constants. As *n*_M_ + *n*_Mp_ + *n*_AMp_ + *n*_AM_=1, one of the states (*n*_M_ in this work) can be assumed dependent on the other three states, leading to a three independent variables system.

### 2.2. Continuous-level model

#### 2.2.1. Contractile unit mechanics

To model the force development within the CU, the work of Murtada *et al.* [17,19,20] is followed. Both phosphorylated and de-phosphorylated attached CB (*n*_AMp_, *n*_AM_) are considered elastic with the same mechanical stiffness. As mentioned previously, *λ*, the current stretch of the CU, can also be defined as the ratio between the current and the reference CU length. The average elastic elongation of the attached CB (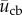) can thus be calculated as follows:
2.20
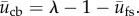
We note that both 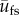 (representing the relative fibre sliding) and 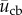 are normalized with respect to the reference CU length and are taken to be negative for contraction. The relative actin–myosin filament sliding 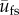 in the CU can be driven either by the myosin power-stroke or the external force/deformation. This internal variable is thus decomposed into a chemical (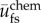) and mechanical (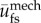) component. The temporal evolution of the chemical component 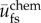 can be derived from the following force balance:
2.21
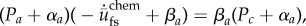
where *P*_*c*_ is the stress associated with the driving force from the cross-bridges while *β*_*a*_ and *α*_*a*_ are fitting parameters. The internal driving stress *P*_*c*_ depends on the contraction/relaxation state of the CU, ie,


2.22
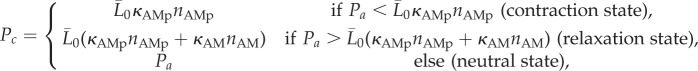


where *κ*_AMp_ is a parameter related to the force of a power-stroke of a single CB and *κ*_AM_ is related to the force-bearing capacity of a dephosphorylated CB during muscle extension. The energy stored in the CU is related to the filament sliding resistance from the surrounding matrix (*P*_*a*_), which can also be seen as the (averaged) first Piola–Kirchhoff stress over the CU:
2.23
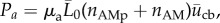
where *μ*_a_ behaves like an active shear modulus and 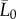 defines the relative filament overlap as a parabolic function of 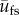:
2.24
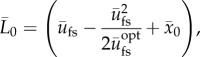
where 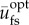 and 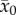 are material parameters.

#### 2.2.2. Tissue structure

From the structural point of view, the medial tissue is considered as a hyper-elastic fibre-reinforced material [11,27], with the fibres aligned along the circumferential direction [20]. Various representations of the arterial tissue have been previously proposed, including models of transverse fibre distribution at a diagonal angle [11,27,32]. These approaches were specifically proposed to accurately represent the adventitia layer of the vessel wall. As we are primarily focusing on the media layer, in this study, we have followed the work proposed in [20] where the media layer fibres are aligned only in the circumferential direction. The free energy function (*Ψ*) is split into volumetric (*Ψ*_vol_) and isochoric components; the latter is then decomposed into active (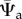), accounting for the CU chemo-mechanics, and passive parts (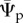), i.e.
2.25
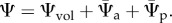
The volumetric part is assumed to be proportional to the energy potential as
2.26
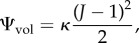
where *κ* and *J* are, respectively, the penalty number and the determinant of the deformation tensor **F** (*J* = det **F**). Both 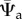 and 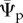 depend on the CU stretch (*λ*), that can also be related to the fourth invariant as
2.27
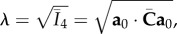
where 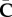 is the deviatoric part of the right Cauchy deformation tensor and **a**_0_ is the direction of the media unstressed fibre. The active component depends directly on the CB attached (*n*_AMp_, *n*_AM_), i.e.
2.28
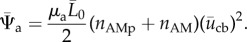
The passive part is modelled as a classical anisotropic material with one fibre aligned with the SMCs as
2.29
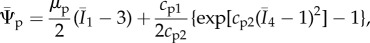
where *μ*_p_, *c*_p1_ and *c*_p2_ are material constants. More details regarding the solid mechanics formulation are provided in electronic supplementary material, S1.

### 2.3. Multiscale coupling and solution procedure

#### 2.3.1. Space discretization

Each level of the framework is discretized by a spatial grid. The cellular network grid reflects the morphology of the tissue, so that each element represents a ‘real’ cell. For the continuous level, a finite-element discretization of the domain was carried out. By assigning each SMC to each finite-element the connectivity of the cellular grid and the mesh coincide. The variables computed at the cellular level (i.e. Ca^2+^ dynamics and CB kinetics) may be considered as internal variables in the finite-element framework. The CU variables (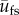, *λ*, etc.) are evaluated at the Gauss integration points of the finite-element, in order to take into account the spatial diversity of deformation over the element. For the structural problem, staggered finite-elements are used in which the displacement field is interpolated linearly while the pressure and dilation coefficient are constant over each element. The nonlinear problem is solved via a classical Newton–Raphson procedure.

#### 2.3.2. Time integration

The models/subsystems constituting the framework are solved in a block segregated fashion, as depicted in figure 1. As there is no feedback between the subsystems, it is possible to employ a different and optimal time integration strategy for each of them. Thus, the Ca^2+^ dynamics is solved by an explicit and adaptive scheme (Runge–Kutta Merson), whereas the time-dependent equations for both the CB kinetics and CU mechanics are solved by the forward Euler method (as in [20]). Note that for computing the CU mechanics the deformed configuration (expressed in terms of *λ*) of the previous time step is used. By comparison, the tissue mechanics problem is solved in an implicit manner. The methodology proposed is valid for either quasi-static or dynamic problems, depending on whether the case considered is under isometric or non-isometric conditions. The same time step was employed for all subsystems. The solution procedure is presented step by step in table 3. The complete model has been implemented into an in-house C++ code. Visualization software ParaView [33] was used for post-processing analysis. The finite-element mesh has been realized by means of the three-dimensional mesh generator software Gmsh [34].

**Table 3.** Solution procedure for evaluating all the system variables in time.

**Cellular level** (solving consecutively two linear problems for *n*_AM_ and *n*_AMp_)
(1) Time initialization *t*=0 (2) Loop over the temporal discretization Δ*τ*_1_ points (3) Loop over the cell framework (the FE mesh in this work) (4) Compute Ca^2+^ variables explicitly (Runge–Kutta Merson) from equations (2.10)–(2.12) (5) Compute rate constant *τ*_1_ from equation (2.17) (6) Compute CB kinetic states explicitly (forward Euler) from equation (2.13)–(2.16) (7) Time update *t* = *t* + Δ*τ*_1_
**Continuous level** (solving a nonlinear problem for *u* and *p*)
(1) Time initialization *t* = 0 (2) Loop over the temporal discretization Δ*τ*_2_ points (3) Newton–Raphson (NR) algorithm (4) Loop over the finite-elements (5) If it is the first NR iteration, then interpolate CB kinetic states in time from Δ*τ*_1_ to Δ*τ*_2_ (6) Loop over the Gauss integration points (7) Compute CB mechano-chemical variables, equations (2.18)–(2.22) (8) Compute first- and second-order derivatives of 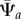 with respect to 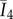 (9) Compute total stress and elasticity tensor (10) Assemble stiffness matrix and residual vector (11) Solve linearized system and variables updating (12) If the residual error condition is satisfied, then time update *t* = *t* + Δ*τ*_2_ and go to 2.

## 3. Experimental study

The pharmacological studies employed in the validation of the modelling methodology along with the model set-up developed to reproduce the experimental settings are reported below.

### 3.1. Experimental protocol

**Figure 4.**
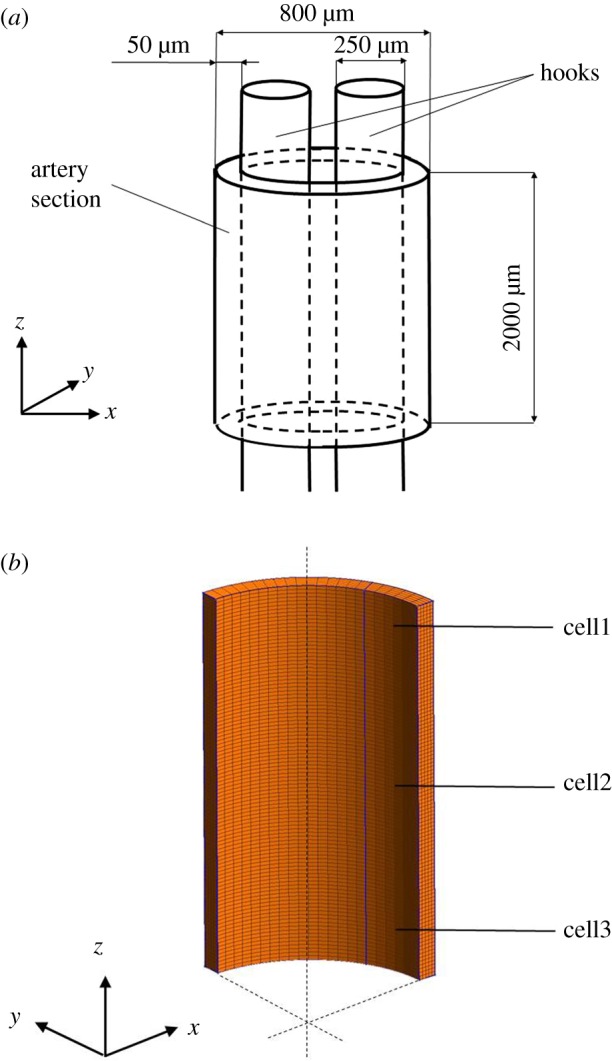
(*a*) Size and geometry of the arterial section set in the myograph through two hooks. (*b*) Location of cell1, cell2 and cell3 in the three-dimensional mesh representing one eighth of the ring. (Online version in colour.)

**Figure 5.**
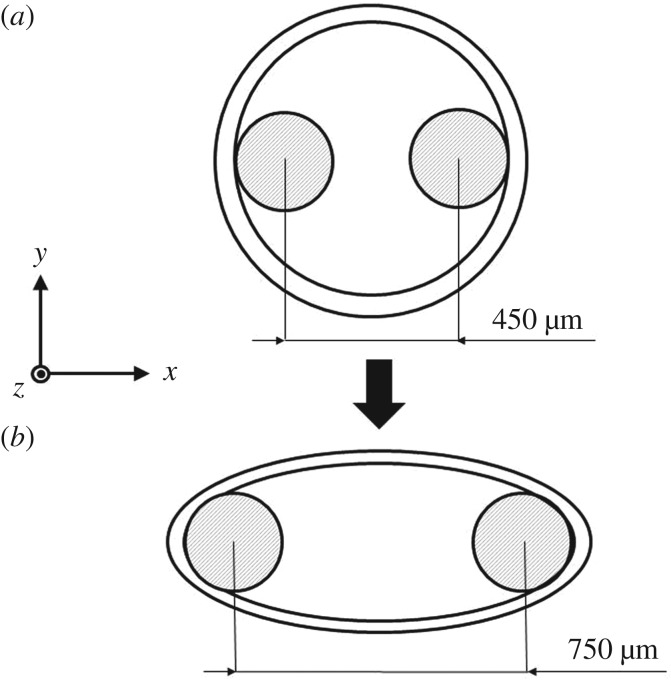
Arterial section deformation during the loading phase, initial (*a*) and stretched (*b*) configurations.

Isolated rabbit ears were obtained as described previously [1], the central ear artery was removed and cleaned of adherent fat and connective tissue. To measure force, 2 mm wide rings were mounted on 0.25 mm diameter steel hooks in a myograph (model 610M, Danish Myotechnology, Aarhus, Denmark) containing oxygenated (95% O_2_; 5% CO_2_) Holman's buffer (composition in mM: NaCl 120, KCl 5, NaH_2_PO_4_ 1.3, NaHCO_3_ 25, CaCl_2_ 2.5, glucose 11 and sucrose 10) at 37.0°C. Prior to any pharmacological interventions, the rings were maintained at a resting tension of 1 mN over a 60 min equilibration period, with frequent readjustments in baseline tension to correct for stress relaxation. The average inner and outer diameters of the annular segments were approximately 0.7 and 0.8 mm, respectively (figure 4*a*). Following the loading phase, the arterial rings obtained the deformed configuration shown in figure 5. By considering a Cartesian reference system, the loading is applied along the *x*-direction. Preparations were incubated for 30 min with both the endothelial nitric oxide synthase inhibitor N(G)-Nitro-l-arginine methyl ester (l-NAME, 300 μM) and the cyclooxygenase inhibitor indomethacin (10 μM) to inhibit prostanoid formation. Rings were then constricted with phenylephrine (PE, 1 μM) and, once constrictor responses had reached a stable plateau, cumulative concentration–response curves to 10 and 30 μM CPA, and 10 and 30 μM ryanodine were obtained.

### 3.2. Model settings

From a structural point of view, each ring is assumed to deform symmetrically with respect to the *x*-plane, while no translations along *y* and in the longitudinal direction (*z*) are expected. The system can thus be reduced to one-eighth of the ring. A finite-element mesh consisting of 5750 linear hexahedral elements is used in the calculations. The global force *F* applied to the hooks is evaluated by integrating the traction contribution of the reduced arterial system over the global contact surface. For the cellular cluster, each cell was associated with one element. At subcellular level, we associate a CU to each Gauss integration point. To account for cellular variability (e.g. size, rates of Ca^2+^ uptake/extrusion) the term corresponding to the influx via Ca^2+^ permeable non-selective cation channels (*Φ*_*A*_) is randomized with a normal distribution (mean value given in table 4 for the relevant pharmacological simulations, and s.d. = 0.1 μM s^− 1^). Randomization of *Φ*_*A*_ creates a population of cells exhibiting variable oscillatory frequency. This allows us to study the effect of increasing strengths of intercellular coupling as a factor of smooth muscle synchronization and generation of vascular tone. For all simulations, the initial values for *χ*, *ζ* and *η* are set equal to 0.1 μM, 0.2 μM and −0.02 V, respectively. The reference set of kinetic rates (*τ*_0_, *τ*_2_, *τ*_3_, *τ*_4_, *τ*_5_) necessary for solving the CB dynamics are taken from Parthimos *et al*. [22].

**Table 4.** Ca^2+^ dynamics and CB kinetics parameters for simulating the drug interventions. The variation of parameters is carried out linearly.

parameter	phenylephrine	CPA	ryanodine
*Φ*_*A*_ (μM s^−1^)	0.6 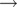 4.2 in Var s	0.8	0.8
*B*_SR_ (μM s^−1^)	400	400 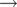 200 in 1000 s	400
*C*_Ry_ (μM s^−1^)	1250	1250	1250 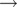 312.5 in 2000 s
*A*_S_ (s^−1^)	0.0	0.0	0.1
*θ*	1.0	30.0	0.5

## 4. Results

### 4.1. Cellular coupling conditions

**Figure 6.**
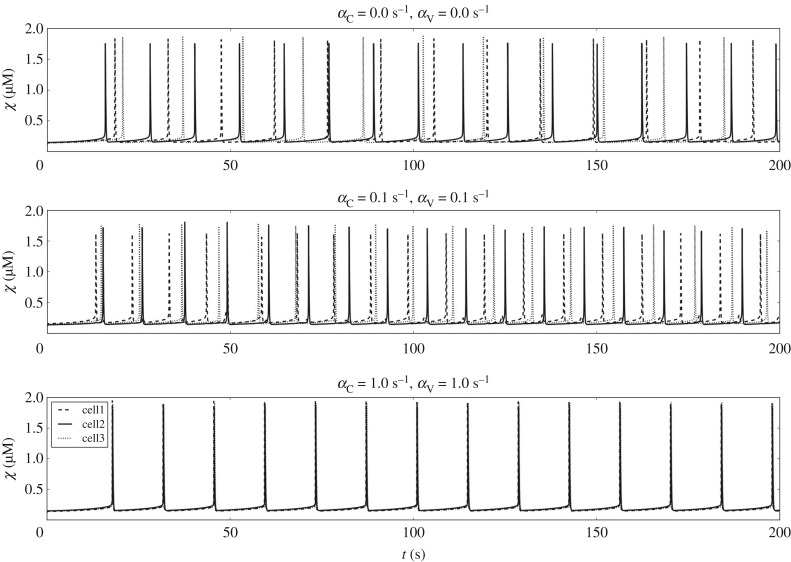
Temporal evolution of *χ* at three different cells (labelled cell1, cell2 and cell3) for different *α*_C_ and *α*_V_.

The proposed analysis is carried out for varying levels of cellular coupling in order to establish the dependency between the diffusion coefficients *α*_C_, *α*_V_ and the global Ca^2+^ dynamics. These simulations are carried out for an unloaded ring configuration. The variables associated with the Ca^2+^ dynamics are monitored for three different cells: cell1, cell2 and cell3 (figure 4*b*). In figure 6, the time evolution of *χ* for (*α*_C_,*α*_V_) equal to 0.0, 0.1, 1.0 s^−1^ is shown. The plot shows clearly that coupling does not affect significantly the amplitude of *χ* signal. The cellular coupling tends to synchronize the *χ* beating pattern along the cluster.

**Figure 7.**
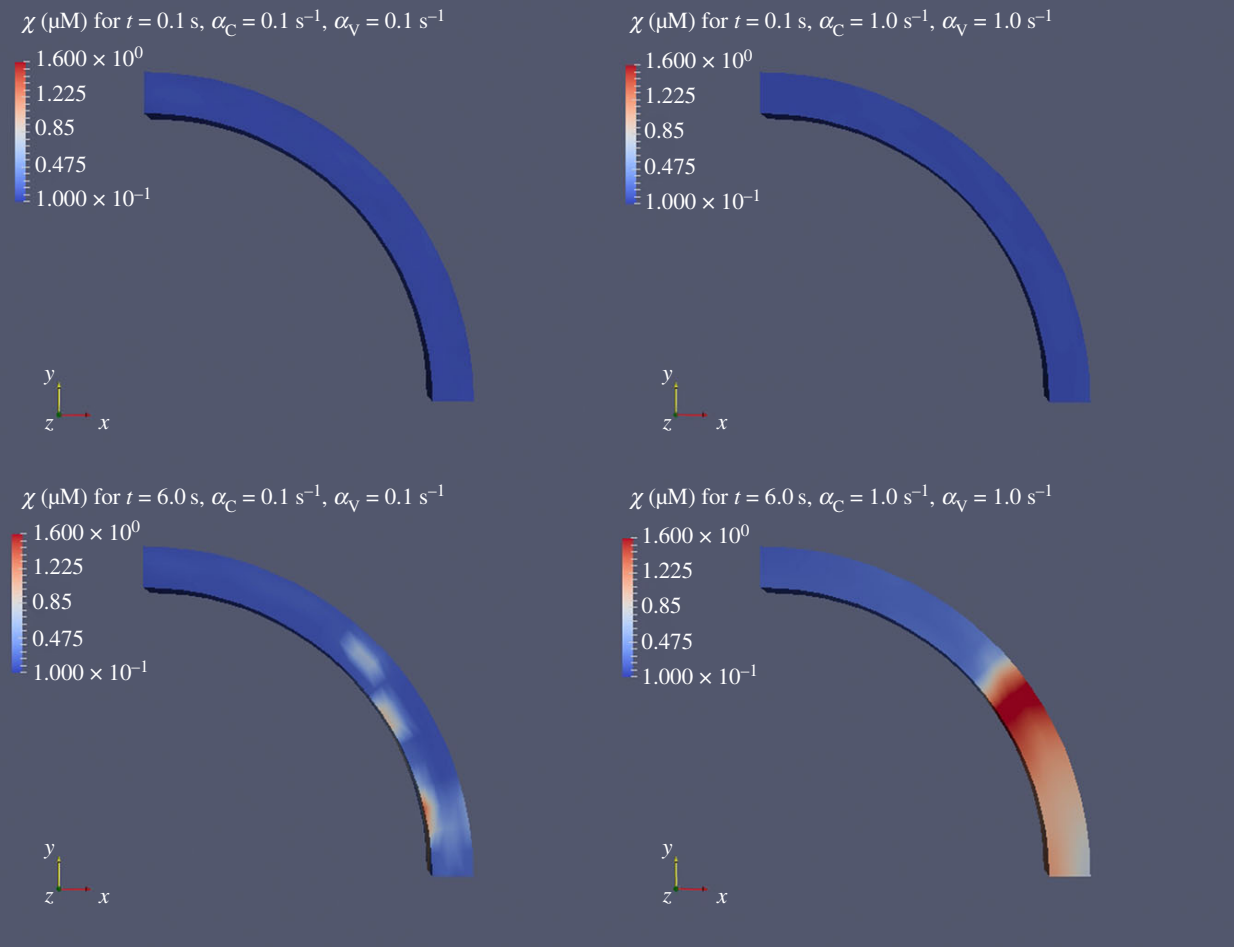
Temporal evolution of *χ* along the spatial domain for (*α*_C_, *α*_V_) = 0.1 s^−1^ and (*α*_C_, *α*_V_) = 1.0 s^−1^. At the top of the figure *χ* is shown at *t* = 0.1 s for an weakly coupled cluster of cells (left) and a strongly coupled one (right). At the bottom part the figure *χ* is shown at *t* = 6.0 s for a weakly coupled cluster of cells (left) and a strongly coupled one (right). The image has been generated by the Paraview software [33].

The spatial distribution of *χ* for two coupling levels (weakly coupled: (*α*_C_, *α*_V_) = 0.1 s^−1^, strongly coupled: (*α*_C_, *α*_V_) = 1.0 s^−1^) at two different time instants (*t* = 0.1 s, *t* = 6.0 s) are shown in figure 7. It is evident that coupling promotes the formation of travelling waves along the annular domain.

### 4.2. Framework validation

A range of pharmacological interventions associated with the activation/inhibition of specific cellular mechanisms was employed for the validation of the model. To mimic the diffusion of pharmacological agents, parametric changes were applied in a graded fashion to all the cells constituting the network. The interventions selected (i.e. phenylephrine, CPA and ryanodine) are associated with the modulation of the cellular Ca^2+^ homeostasis which is reflected in the contractile state of the smooth muscle. The actions of these pharmacological probes were simulated by the gradual variations of the associated model parameters, reported in table 4. The actin and myosin filaments are assumed to be detached for *t* = 0 s (*n*_M_ = 0.5 and *n*_Mp_ = 0.5), while the initial 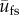 is set equal to 0 for each CU. Cells are assumed to be strongly coupled with (*α*_C_,*α*_V_) set equal to 1.0 s^−1^. The simulated drug interventions were performed only after the Ca^2+^ and CB variables reached stationary conditions. For each plotted result the drug intervention occurred at *t* = 0 s unless otherwise stated.

As the ring is clamped at the hooks, the resultant sum between internal and external forces at the nodes in contact with the hooks must be null. In this study, we are mainly interested in the global force developed at the hooks, which can be seen as the sum of nodal contributions along the contact surface. In addition to this, we compute also the force developed locally at cell1, which is assumed to be proportional to the sum *n*_AMp_+*n*_AM_ [22]. For a more complete view of how the contractile force emerges from Ca^2+^ dynamics at the cellular level, see electronic supplementary material, S2.

#### 4.2.1. Phenylephrine intervention

**Figure 8.**
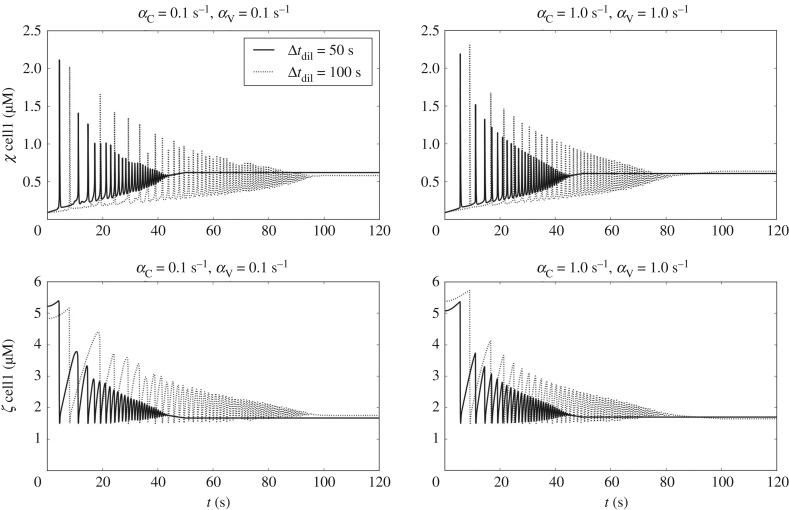
Time evolution of *χ* and *ζ* (cell1) for a simulated phenylephrine intervention. Results are shown for different drug dilution times (Δ*t*_dil_ = 50 and 100 s) and coupling conditions ((*α*_C_, *α*_V_) = 0.1 s^−1^ and (*α*_C_, *α*_V_) = 1.0 s^−1^).

Phenylephrine is a selective agonist of *α*1-adrenergic receptor, associated with vasoconstriction. The action of phenylephrine at cellular level was modelled by increasing the cytosolic Ca^2+^ influx via NSCCs. A more complete picture of the action of phenylephrine involves an initial Ca^2+^ release from the SR, via inositol 1,4,5-trisphosphate-sensitive Ca^2+^ release channels (IP3R channels), followed by sustained Ca^2+^ influx into the cytosol through NSCCs. To simulate the action of phenylephrine, the coefficient *Φ*_*A*_ of each individual cell was increased by a factor of 7. Therefore, the mean value of the *Φ*_*A*_ distribution increased from 0.6 to 4.2 μM s^−1^. Two different drug dilution times (Δ*t*_dil_ = 50 and 100 s) were used, as shown in figure 8. By increasing cytosolic Ca^2+^ uptake, *χ* in cell1 starts to oscillate periodically. This pattern is reflected also in the store Ca^2+^ concentration variations (figure 8). As expected, higher Δ*t*_dil_ involves longer transient before reaching stationary conditions.

**Figure 9.**
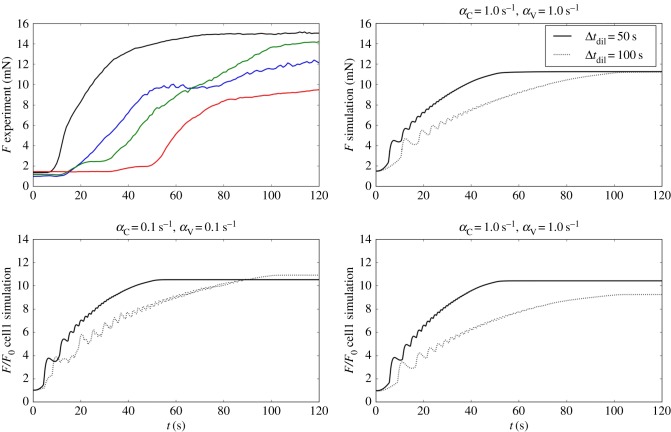
Time evolution of the experimental and simulated forces at the hooks for a simulated phenylephrine intervention. Experimental forces (coloured lines) are plotted for four different ring measurements. The theoretical results are shown for different drug dilution times (Δ*t*_dil_ = 50 and 100 s) and coupling conditions ((*α*_C_, *α*_V_) = 0.1 s^−1^ and (*α*_C_, *α*_V_) = 1.0 s^−1^). The cellular forces values are normalized with respect to the initial force *F*_0_. (Online version in colour.)

The forces generated by this intervention, normalized with respect to the force developed at the beginning of the intervention, are presented in figure 9, where three responses are shown for different dilution times (Δ*t*_dil_) and different cellular coupling (*α*_C_, *α*_V_). By comparison to the simulated results, it appears that the drug was able to activate the muscle tissue at a dilution time Δ*t*_dil_ ∼ 50 s. We also observe that the final magnitude of the simulated and measured force is very similar.

#### 4.2.2. Cyclopiazonic acid intervention

**Figure 10.**
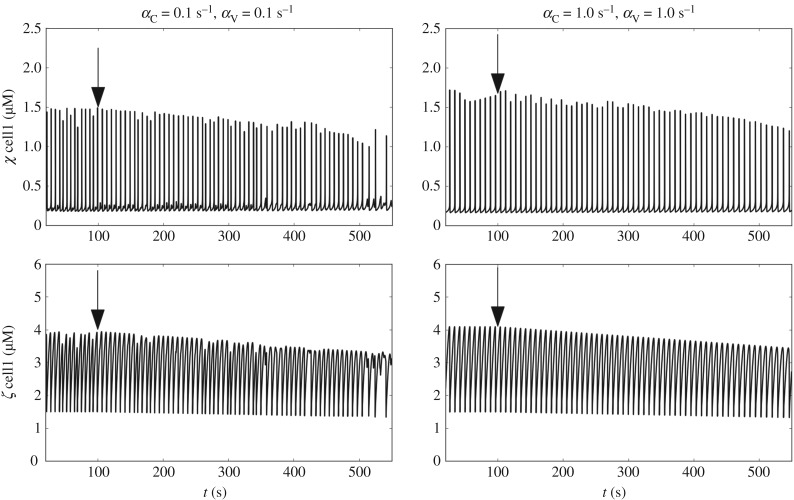
Time evolution of *χ* and *ζ* (cell1) for a simulated CPA intervention for different coupling conditions ((*α*_C_, *α*_V_) = 0.1 s^−1^ and (*α*_C_, *α*_V_) = 1.0 s^−1^). Arrows show the intervention time.

**Figure 11.**
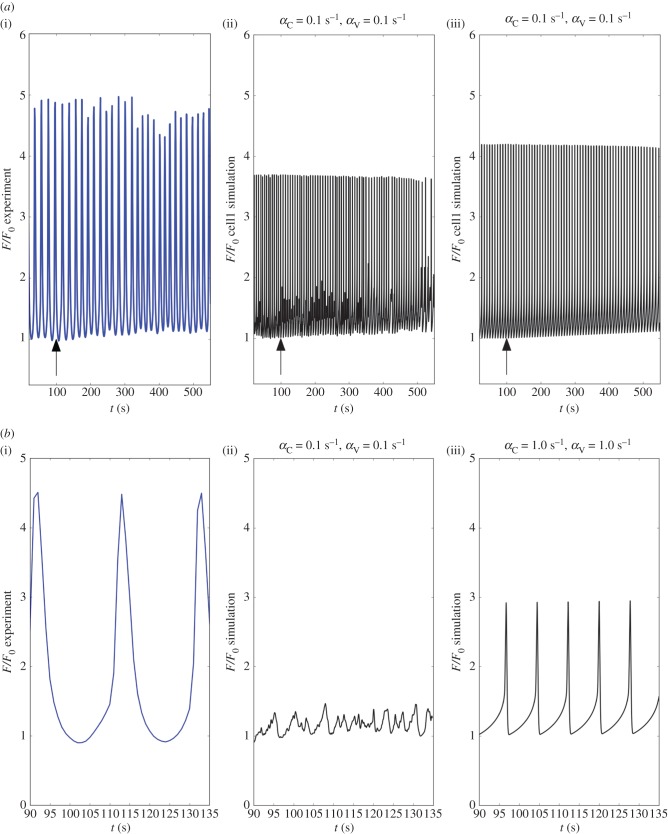
Time evolution of the experimental and simulated forces at the hooks for a simulated CPA intervention for different coupling conditions ((*α*_C_, *α*_V_) = 0.1 s^−1^ and (*α*_C_, *α*_V_) = 1.0 s^−1^). (*a*) shows the comparison between the experimental force recorded in time, in the presence of 30 μM CPA, against the simulated singular cellular force responses. Arrows show the intervention time. In (*b*) the differences between experimental force and (global) simulated force patterns are shown. The plotted values are normalized with respect to the initial force *F*_0_. (Online version in colour.)

Cyclopiazonic acid (CPA) is an inhibitor of the SERCA pump, preventing refilling of the store and is thus associated with Ca^2+^ store depletion. The effect of CPA, in terms of modulating the intracellular Ca^2+^ oscillator, is highly concentration-dependent as shown previously [35]. CPA was administered through concentration increases from 10 to 30 μM. In a few experiments CPA concentrations of 100 μM were used, which accelerated the tapering effect already in evidence (see electronic supplementary material, S2). For simulation purposes, the action of CPA was reproduced by linearly decreasing coefficient *B*_SR_ from 400 to 350 μM in 2000 s (while the randomized coefficient *Φ*_*A*_ was maintained constant for each cell, with a mean distribution value of 0.8 μM s^−1^). In both the experiment and simulations CPA caused a small reduction in the oscillatory amplitude, while a regular waveform was maintained throughout the simulated intervention (figure 10). A comparison between the experimental and simulated time series is shown in figures 11*a*,*b*. Note that the oscillatory amplitude of a SMC weakly coupled to its neighbours is reduced, when compared with the same cell under strongly coupled conditions (figure 11*a*(ii)(iii)). This effect of weak cell–cell coupling is greatly enhanced in the case of force development, as seen in figure 11*b*. This is due to the fact that the force contributions of individual cells remain largely uncoordinated. Global force development is restored under strong intercellular coupling conditions (figure 11*b*(iii)).

#### 4.2.3. Ryanodine intervention

**Figure 12.**
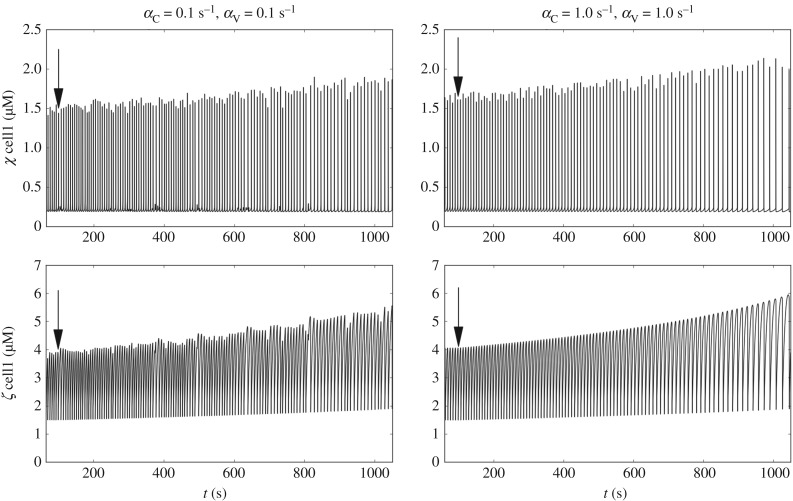
Time evolution of *χ* and *ζ* (cell1) for a simulated ryanodine intervention for different coupling conditions ((*α*_C_, *α*_V_) = 0.1 s^−1^ and (*α*_C_, *α*_V_) = 1.0 s^−1^). Arrows show the intervention time.

Ryanodine was administrated at the arterial sample at concentrations of 10 and 30 μM. As discussed previously, the action of ryanodine can be simulated in different ways depending on the concentration [22,23]. This is due to the complex multistage configuration of the ryanodine receptor tetramer. For the concentrations of ryanodine used in this study, it is accepted that the compound will block Ca^2+^ release from the SR in a concentration-related fashion. The action of ryanodine was simulated according to [22], by linearly decreasing the coefficient *C*_Ry_ at *t* = 100 s from 1250 μM s^−1^ down to 312.5 μM s^−1^. The temporal evolution of variables *χ* and *ζ* within the reference cell1 is shown in figure 12 for different cellular coupling conditions ((*α*_C_, *α*_V_) = 0.1 s^−1^ and (*α*_C_, *α*_V_) = 1.0 s^−1^). Gradual attenuation of Ca^2+^ release via the ryanodine receptor channels is associated with a decrease in frequency.

**Figure 13.**
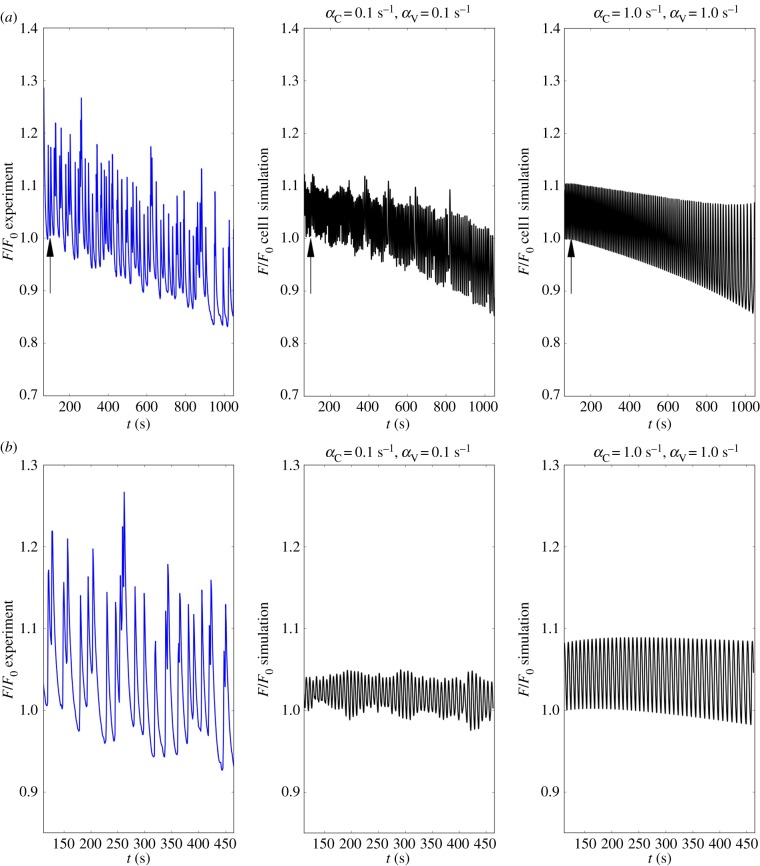
Time evolution of the experimental and simulated forces at the hooks for a simulated ryanodine intervention for different coupling conditions ((*α*_C_, *α*_V_) = 0.1 s^−1^ and (*α*_C_, *α*_V_) = 1.0 s^−1^). (*a*) Shows the comparison between the experimental force recorded in time, in the presence of 10 μM ryanodine, against the simulated singular cellular force responses. Arrows show the intervention time. In (*b*), the differences between experimental force and (global) simulated force patterns are shown. The plotted values are normalized with respect to the initial force *F*_0_. (Online version in colour.)

The forces obtained from the model are compared against the experimental values in figure 13*a*,*b*. In this case, coefficient *θ* was equal to 0.5. The pattern of the forces follows Ca^2+^ concentrations, with the same gradual decreasing magnitude. To simulate this aspect of the experimental traces, we needed to employ the term in equation (2.1) associated with store-operated Ca^2+^ entry, which is triggered in response to levels of SR Ca^2+^. Note that this mechanism only had a minor effect in the simulation of the phenylephrine and CPA responses. Similar to the CPA simulations, weakly coupled SMCs remain uncoordinated in the presence of ryanodine, producing weak global contractile force (figure 13*b*). A consistent vascular tone is restored when strong intercellular coupling is maintained.

## 5. Discussion

We have developed a multi-component mathematical modelling framework that accounts for the structural response of the arterial wall under the active contraction of the media smooth muscle layer. The methodology was validated against a set of pharmacological interventions that modulate the contractile apparatus at the cellular level. The multiscale modelling approach combines dynamics and mechanics occurring at different levels, each requiring a specific solution strategy. With regard to the mechano-elastic component, the study was performed under isometric conditions, and thus the inertial force is neglected, allowing us to deal with a simplified system in which the evaluation of a number of dynamic parameters (such as density) was not necessary. Moreover, the ability to choose a finite-element discretization that conforms to the cellular grid, eliminated the need for spatial and temporal interpolation between the two subsystems. As a consequence, it was possible to adopt the same time step for all elements of the model. This is not a limitation, as different integration steps can be selected for each model level if required by the specific problem. This strategy can be implemented in conjunction with interpolation techniques that allow information transmission between the various contributing systems. Although necessary in many cases, this approach can result in loss of accuracy. All experiments in this study were performed following administration of l-NAME to eliminate the inhibitory effect of endothelium-derived nitric oxide on the smooth muscle contractile apparatus [36]. The involvement of secondary endothelium produced electrochemical factors in the contractile activity of the arterial wall has not been included, and should form the basis for further elaboration of the current model [37]. Several arterial ring experiments under isometric conditions have been carried out, each involving four rings ≈ 2 mm in length excised from the same animal. In spite of efforts to maintain identical conditions, variability was present, particularly between individual animals. With respect to the four arterial rings from the same arterial sample (e.g. figure 9), experimental and biological variability that could not be eliminated was due to differences in length, diameter (all rings were from adjacent sections, but there is, inevitably, a reduction in diameter as you descend the vessel), and purely initial condition considerations, such as Ca^2+^ and ionic uptake levels, which have been shown both theoretically and experimentally to greatly affect the oscillatory response of the arterial wall [22,23,30]. Owing to these factors, each arterial ring is at a different initial contractile state, reflected in variable unloaded geometry. We therefore elected to normalize the force traces against the pre-intervention steady state (e.g. the pre phenylephrine force in figure 9) which would allow us to reproduce the percentile contractile response during pharmacological probing. This approach successfully facilitated the main focus of this work which was the integration of the smooth muscle-based contractile apparatus and the mechano-elastic properties of the arterial wall. To investigate this fundamental interaction in the genesis of arterial tone, we have employed a series of experimental studies that probe distinct aspects of these mechanisms [22,23]. By simulating experimental findings with the vasoconstrictor phenylephrine, we were able to match the response time of the drug, and demonstrate the direct link between cellular Ca^2+^ dynamics and the development of force at the tissue level. Indeed, the cellular events occur at the same timescale as the global tissue contraction. This finding is correlated to the increased cytosolic Ca^2+^ concentrations associated with the administration of phenylephrine. This observation highlights the central role of cytosolic Ca^2+^ influx in the generation of vascular tone and the onset of oscillatory activity observed in both experiments and simulations. Indeed, we have previously shown that the levels of Ca^2+^ influx can modulate other ionic signalling pathways in a specific, clinically relevant manner [22]. As noted earlier, phenylephrine mediates Ca^2+^ release through the IP3R channel pathway. We have previously employed different approaches to account for this effect. In [22], we have considered two independent CICR and InsP3-induced Ca^2+^ (IICR) release pathways and assumed that the IICR results in a constant Ca^2+^ release current, which is essentially lumped in the overall Ca^2+^ uptake by the cell. Both effects are therefore accounted for by the same constant *Φ*_*A*_. We have shown that such an assumption still allowed the mathematical model of vasomotion to accurately reproduce an extensive range of pharmacological interventions [22]. Further work to distinguish the CICR from the IICR mechanism reproduced the same results with no increased accuracy. We have therefore assumed here the simplified scenario proposed in [22]. The potential of Ca^2+^ influx to determine the natural frequency of the cell's contractile apparatus was the main reason that parameter *Φ*_*A*_ was selected for randomization across the cellular population. By comparison, cellular dynamics are considerably less sensitive to variations of other system parameters, associated with alternative ionic fluxes [29]. Blockage of the SERCA pump with CPA resulted in a modest pattern variation that highlights the robustness of the store-refilling mechanism. The resilience of Ca^2+^ dynamics under CPA was reproduced by the simulations. A noteworthy aspect of the function of CPA is its ability to transform arterial vasomotion in a controlled manner that follows hallmark transition routes out of chaotic behaviour [35,38]. Evidence of this behaviour is shown in figure 11*a*,*b* although detailed investigation of nonlinear oscillatory transitions was not an aim of this work. Ryanodine receptor dysregulation is implicated in a range of neuromuscular disorders and arrhythmogenesis in cardiovascular diseases [3]. This is mainly due to the complex inter- and intra-subunit interactions within the ryanodine receptor homotetramer [4,5]. Considering the intricate multistage dynamics of the ryanodine channel, computational simulation work can elucidate some of the dominant components of the mechanism. Although studied here in isolation, dysregulation of the ryanodine channel has been associated with upregulation of the SERCA pump protein, to allow for the maintenance of a level of sustainable homeostasis [39,40]. Such findings support the proposed methodology as a testing ground for hypotheses on the pathogenesis of vascular disease.

## Supplementary Material

Theoretical Formulation

## Supplementary Material

Experimental and Simulation Results

## Data accessibility

Further experimental data and numerical results supporting this article have been uploaded as part of electronic supplementary material, S2.
