# Supplementary material for: A multiscale active structural model of the arterial wall accounting for smooth muscle dynamics
Source: J R Soc Interface. 2018 Feb 7;15(139):20170732. doi: 10.1098/rsif.2017.0732 (PMC5832725; doi:10.1098/rsif.2017.0732)
Supplement: Theoretical Formulation [file rsif20170732supp1.pdf]

Supplementary Material 1: Solid mechanics formulation

Variational principle for solid tissue

In order to prevent volmetric locking a three field Hu-Washizu formulation has been used. The functional depends on the state of deformation (ϕ), the volume change indipendedently of the motion ($\bar{J}$) and the pressure (p) that is a Lagrange multiplier enforcing the condition that $J = \bar{J}$. The framework is split into deviatoric and volumetric components

$$\Pi_{HW}(\phi, \bar{J}, p) = \int_V \hat{\Psi}(\mathbf{C})dV + \int_V U(\bar{J})dV + \int_V p(J - \bar{J})dV - \Pi_{ext}(\phi) \tag{1}$$

where $\mathbf{C}$ is the right Cauchy deformation tensor.

The stationary conditions of Equation (1) with respect to $\bar{J}$,ϕ and p will yield the virtual work principle and the constitutive and kinematic relationships associated with the volumetric behavior:

$$\begin{cases} D\Pi_{HW}(\phi, \bar{J}, p)[\delta \boldsymbol{v}] = \int_v \boldsymbol{\sigma} : \delta \mathbf{d}\, dv - \delta W_{ext}(\phi, \delta \boldsymbol{v}) = 0 \\ D\Pi_{HW}(\phi, \bar{J}, p)[\delta \bar{J}] = \int_V (\frac{dU}{d\bar{J}} - p)\delta \bar{J}\, dV = 0 \\ D\Pi_{HW}(\phi, \bar{J}, p)[\delta p] = \int_V (J - \bar{J})\delta p\, dV = 0 \end{cases} \tag{2}$$

where J is the pointwise jacobian such that $J = \frac{dv}{dV}$. As $\delta \bar{J}$ and δp can be any arbitrary functions, Equation (2) gives $\bar{J} = J$ and $p = \frac{dU}{d\bar{J}}$.

Cauchy deformation and stress tensors

The Right Cauchy Deformation Tensor is calculated as

$$\mathbf{C} = \mathbf{F}^T \mathbf{F} \tag{3}$$

The Cauchy Stress Tensor is computed as

$$\sigma = \sigma_{vol} + \bar{\sigma}_a + \bar{\sigma}_p \tag{4}$$

where:

$$\sigma_{vol} = \kappa \frac{J-1}{J} \mathbf{I} \tag{5}$$

$$\bar{\sigma}_a = \frac{2}{J} \frac{d\bar{\Psi}_a}{d\bar{I}_4} \mathrm{dev}(\mathbf{a} \otimes \mathbf{a}) \tag{6}$$

$$\bar{\sigma}_p = \frac{\mu_p}{J} \mathrm{dev}\bar{\mathbf{b}} + \frac{2}{J} \frac{d\bar{\Psi}_p}{d\bar{I}_4} \mathrm{dev}(\mathbf{a} \otimes \mathbf{a}) \tag{7}$$

with:

$$\frac{d\bar{\Psi}_a}{d\bar{I}_4} = \mu_a \bar{L}_0 (n_{AMp} + n_{AM}) \frac{\lambda - 1 - \bar{u}_{fs}}{2\bar{I}_4} \tag{8}$$

$$\frac{d\bar{\Psi}_p}{d\bar{I}_4} = c_{p1}(\bar{I}_4 - 1)\exp\left[c_{p2}(\bar{I}_4 - 1)^2\right] \tag{9}$$
