# Supplementary material for: A multiscale active structural model of the arterial wall accounting for smooth muscle dynamics
Source: J R Soc Interface. 2018 Feb 7;15(139):20170732. doi: 10.1098/rsif.2017.0732 (PMC5832725; doi:10.1098/rsif.2017.0732)
Supplement: Experimental and Simulation Results [file rsif20170732supp2.pdf]

Supplementary material 2: Cellular dynamics

Three scenarios of cellular Ca^{2+} homeostasis and contractile response, for parameters Φ_A=1, 2 and a transition from 1→ 2. As stated in the manuscript, parameter Φ_A, corresponding to a net Ca^{2+} influx into the cytosol, has a profound effect on the frequency of oscillatory response.

Results provided are for a single cell (i.e. no intercellular coupling).

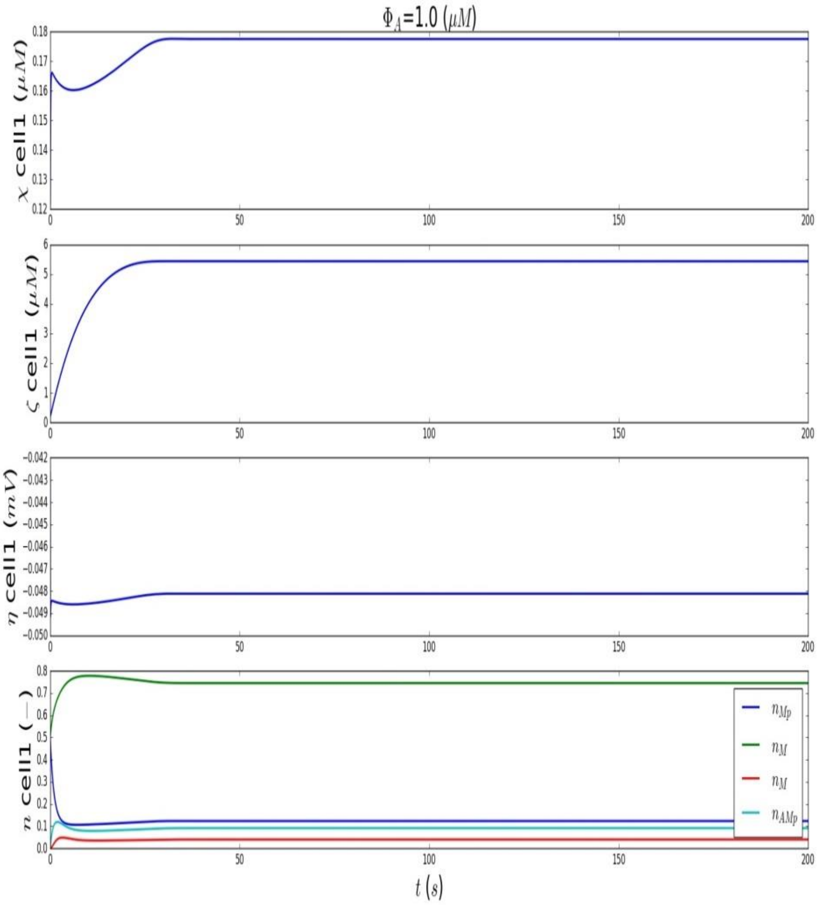

Figure S1. Time evolution of cytosolic Ca^{2+} concentrations (χ), Ca^{2+} in the sarcoplasmic reticulum (ζ), and membrane potential (η) for Φ_A=1 (upper three panels). Lower panel presents the four components of the contractile unit response. The concentrations reach a steady-state plateau within 30 seconds. Note that no oscillations occur as the Ca^{2+} influx is insufficient to trigger the CICR mechanism.
Parameters employed: dt=10^{-2}s, A_S=0, B_1=400, E_{Ca}=12, E_k=43. Other parameters as in Tables 1 and 2. α_c=α_v=0, i.e. no intercellular coupling.

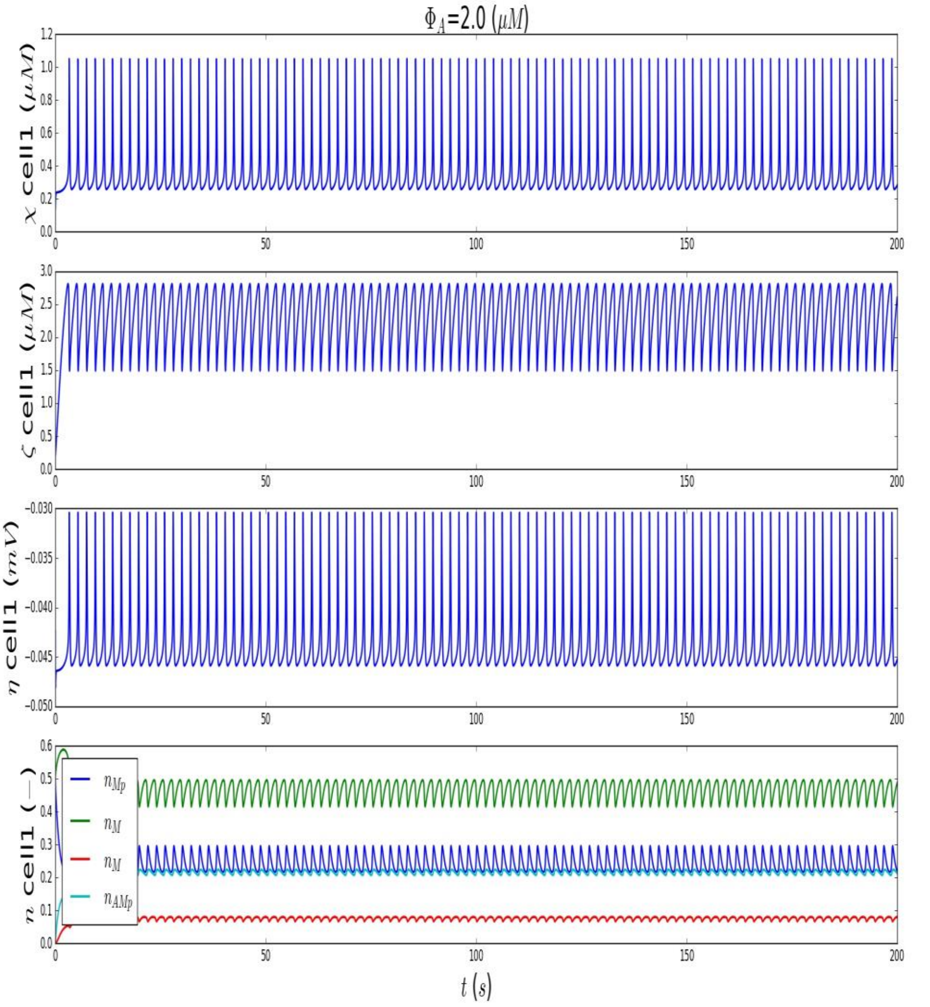

Figure S2. Time evolution of cytosolic Ca^{2+} concentrations (χ), Ca^{2+} in the sarcoplasmic reticulum (ζ), and membrane potential (η) for Φ_A=2 (upper three panels). Lower panel presents the four components of the contractile unit response. All traces are in an oscillatory range, as Ca^{2+} influx is adequate to initiate and sustain the Ca^{2+}-induced Ca^{2+} release from intracellular stores. Note that transition phase is very short at <10s. Parameters are the same as in the previous simulation.

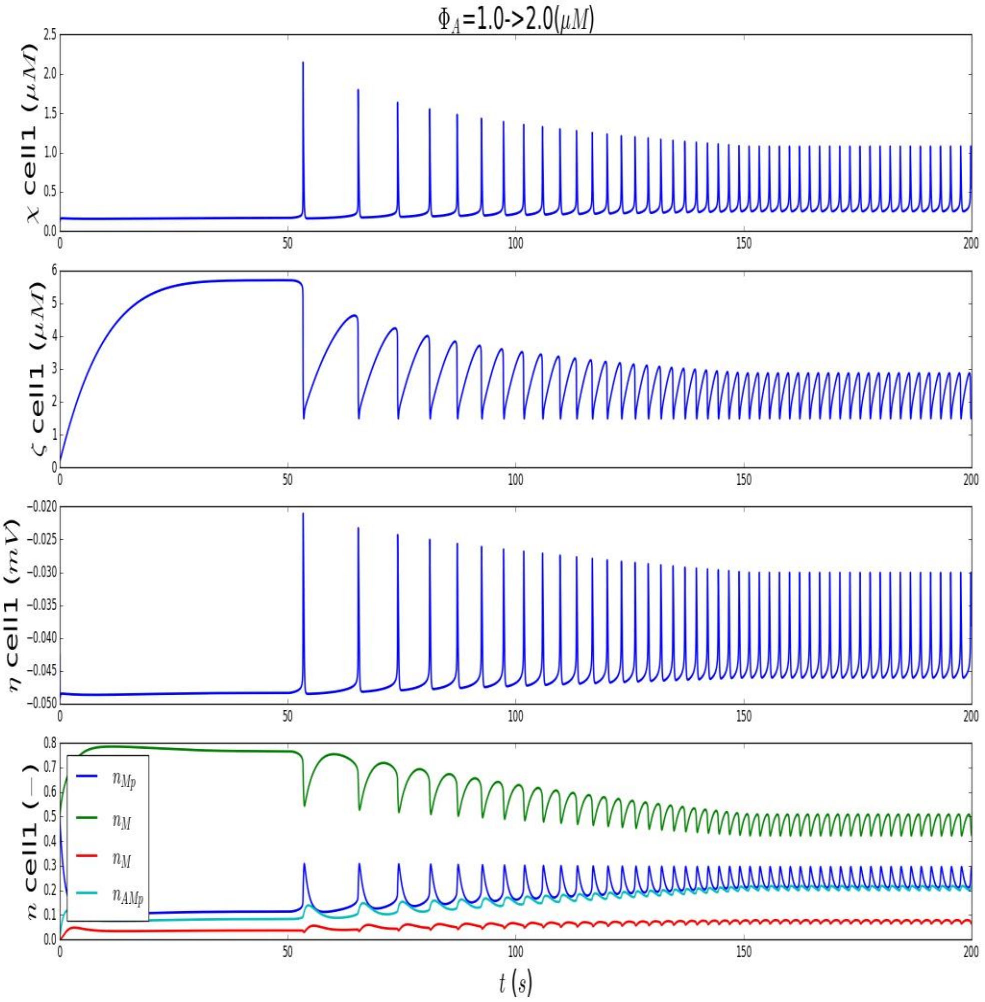

Figure S3. Time series of variables described in the previous two Figures. Simulations correspond to a transition of variable Φ_A from 1 → 2, illustrating the initiation of oscillatory activity. The transition phase lasted from from 50 to 100 s.

Simulation parameters same as in Figures S1, S2.

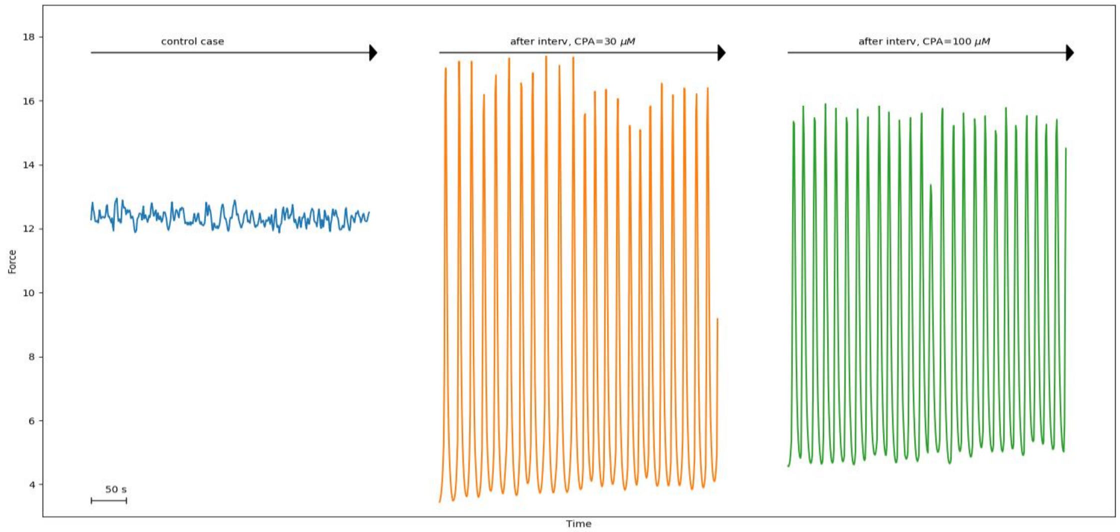

Figure S4. Experimental time-series: Force evolution during CPA interventions. The three segments correspond to Control (pre-intervention), 30 μM and 100 μM CPA concentrations.

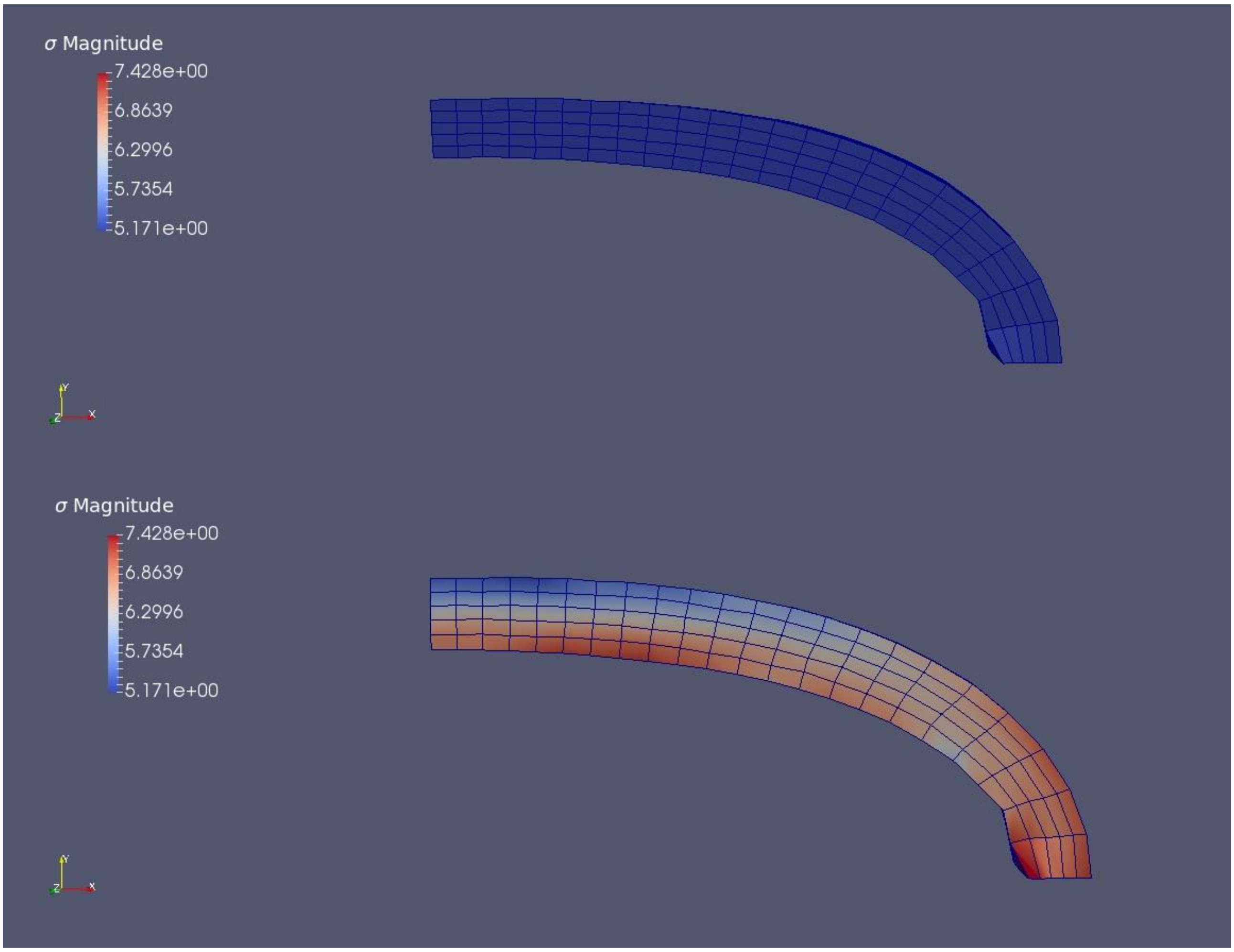

Figure S5. Stress distribution along the arterial ring before and after simulated phenylephrine intervention. The quantity plotted has been post-processed by the software ParaView (A. Henderson, ParaView Guide, A Parallel Visualization Application. Kitware Inc., 2007), option "Magnitude".
